# Emerging adults’ intersecting experiences of food insecurity, unsafe neighbourhoods and discrimination during the coronavirus disease 2019 (COVID-19) outbreak

**DOI:** 10.1017/S136898002000422X

**Published:** 2020-10-23

**Authors:** Nicole Larson, Jaime Slaughter-Acey, Tricia Alexander, Jerica Berge, Lisa Harnack, Dianne Neumark-Sztainer

**Affiliations:** 1 Division of Epidemiology and Community Health, School of Public Health, University of Minnesota, Suite 300, 1300 South Second Street, Minneapolis, MN 55454, USA; 2 Department of Family Medicine and Community Health, University of Minnesota, Minneapolis, MN, USA

**Keywords:** Food insecurity, Home food availability, Neighbourhood safety, Discrimination, Emerging adult

## Abstract

**Objective:**

To examine how food insecurity is related to emerging adults’ food behaviours and experiences of neighbourhood safety and discrimination and to identify resources needed to support their health during the COVID-19 outbreak.

**Design:**

Rapid response online survey. Participants completed the six-item US Household Food Security Survey Module, a brief measure of food insufficiency, and measures of food behaviours, neighbourhood safety and discrimination. Open-ended questions were used to assess changes in eating behaviours during COVID-19 and needed resources.

**Setting:**

C-EAT (COVID-19 Eating and Activity over Time) study invitations were sent by email and text message to a longitudinal cohort.

**Participants:**

A total of 218 emerging adults (mean age = 24·6 (sd 2·0) years, 70·2 % female) completed a survey in April–May 2020 during a stay-at-home order in Minnesota.

**Results:**

The past year prevalence of food insecurity was 28·4 %. Among food-insecure respondents, 41·0 % reported both eating less and experiencing hunger due to lack of money in the past month. Food-insecure respondents were less likely than those who were food secure to have fruits/vegetables at home and more likely to have frequent fast-food restaurant meals, feel unsafe in their neighbourhood and experience discrimination during the stay-at-home order. Food-insecure adults reported changes including eating more food prepared at home, eating more take-out restaurant meals and purchasing more energy-dense snacks as a result of events related to COVID-19. Resources most needed to support their health included eligibility for more food assistance and relief funds.

**Conclusions:**

Food-insecure emerging adults experience many barriers to maintaining healthful eating patterns during COVID-19.

Having a safe and dignified manner to access adequate food is a basic human right. The condition of food insecurity (i.e. lacking reliable access to adequate food for an active and healthy life) is associated with poorer health behaviours and compromised physical and mental well-being^(1–7)^. Federal and local policies and programmes that support equitable access to adequate food are critical to ensuring the health of populations and especially vulnerable groups impacted by public health emergencies such as the COVID-19 pandemic. The life course transition from adolescence to adulthood is a period of particular vulnerability for experiencing food insecurity and its impacts on health^(8,9)^.

A 2018 survey of the EAT 2010–2018 (Eating and Activity over Time) study cohort from Minneapolis–St. Paul, Minnesota found that about one in four emerging adults (18–26 years) had experienced food insufficiency in the past year^(8)^. The subgroups of emerging adults who reported unemployment, living with children of their own and an ethnic/racial identity of African American, Black or Native American were most likely to have experienced food insufficiency^(8)^. These observed disparities among the emerging adults who were invited to participate in the present study are in line with disparities identified by national surveillance data for the overall US population^(10)^. The disparities highlight the need for more attention to structural and racial barriers to health and their influence on susceptibility to food insecurity^(11,12)^. Only a small number of studies have investigated linkages between neighbourhood safety, exposure to racism and experiencing food insecurity; however, the available evidence suggests that unsafe neighbourhood conditions and multiple forms of experiencing discrimination are likely to have substantial negative impacts on one’s ability to access adequate food^(13–16)^.

The US outbreak of COVID-19 has intensified challenges and disparities in food access among vulnerable populations and led to an epidemic of food insecurity^(17)^. National survey data indicate that the prevalence of food insecurity has recently doubled in the overall population and tripled among households with children as a result of many businesses and schools being forced to shut^(18)^. The stay-at-home orders that were implemented as part of efforts to protect public health have led to increasing unemployment and new barriers to food access^(19–21)^. Several recent changes in the operation of food assistance programmes (e.g. grocery deliveries by food shelves) and supportive policies (e.g. Families First Coronavirus Response Act) have been enacted to respond to the crisis^(22)^. There is however little known about home food availability and food shopping and eating behaviours during COVID-19 with specific concern for households that have experienced food insecurity at one or more times in the past year. In order to guide policy decisions about food assistance programmes and supportive services, and the design of effective health promotion messaging for public health emergencies, it will be important to build understanding of the food behaviours and discrimination experiences of those who are faced with food insecurity.

As one of the first US studies to examine food insecurity and related factors during COVID-19^(17,23–25)^, the present study was specifically designed to help inform the development of policies and services for emerging adults. The three aims focus on food insecurity and intersecting problems. The first aim is to describe how past year experiences of food insecurity are related to emerging adults’ experiences of food insufficiency, food shopping behaviours, home food availability, eating behaviours and experiences of neighbourhood safety and discrimination during COVID-19. Secondly, the study examines how food shopping, food preparation and eating behaviours have been influenced by events related to COVID-19 for emerging adults who were food insecure at one or more times in the past year. The third aim is to identify the resources that are most needed by food-insecure emerging adults to help them gain access to healthy foods and maintain their overall health during a public health emergency.

## Methods

### Study design and sample

Participants in the C-EAT (COVID-19 Eating and Activity over Time) study were members of the EAT 2010–2018 longitudinal study cohort and invited to complete a follow-up online survey in the spring of 2020 during the initial months of the US outbreak of COVID-19. The EAT 2010–2018 study is a population-based investigation of weight-related health behaviours and associated factors among 1568 young people who were attending secondary school in Minneapolis–St. Paul, Minnesota in 2009–2010^(8,26)^. Invitations to participate in the online C-EAT survey were sent to cohort members by email and text message in April 2020. A reminder message was similarly sent to a subset of non-responders who had provided both forms of contact information. Responses to the survey had been received from 218 young people (21 % of those who could be contacted) at the time that COVID-19 emergency stay-at-home orders were lifted in Minnesota on 18 May. All participants were mailed a financial incentive of $25 following survey completion. The University of Minnesota Institutional Review Board Human Subjects Committee approved all protocols.

The C-EAT survey sample included 153 females, 62 males and 3 participants indicating another sex identity. The sample had a mean age of 24·6 (sd 2·0) years in 2020. Although C-EAT survey participants were less likely than 2018 survey participants to identify as male, identify their race as African American or Black and have a parent of lower SES, C-EAT participants were of diverse backgrounds. The distribution of ethnic/racial backgrounds reported by C-EAT participants was similar but more diverse than the overall population in Minneapolis–St. Paul, Minnesota with 36·4 % White, 19·4 % Asian American, 19·3 % Hispanic, 14·3 % African American or Black and 10·6 % mixed or other^(27)^. The distribution across categories of parental SES based primarily on baseline educational attainment was: 30·3 % low, 16·1 % low-middle, 18·3 % middle, 21·1 % upper-middle and 14·2 % high. Most participants (87 %) were living in Minnesota at the time they completed the C-EAT survey.

### Survey measures

The C-EAT survey was based on prior EAT surveys with modifications made to focus recall on the past month of events related to the COVID-19 outbreak^(28)^. The survey measures included a six-item validated tool for assessing food insecurity in the past year^(29)^. Two additional items were asked to assess experiences of food insufficiency in the past month. If participants responded ‘yes’ when asked ‘did you ever eat less than you felt you should’ and also ‘yes’ when asked ‘were you ever hungry but didn’t eat’ because ‘there was not enough money for food’ in the past month, they were categorised as food insufficient. The C-EAT survey was the source of nearly all measures described in Table 1 (including food shopping behaviours, home food availability, eating behaviours and experiences of neighbourhood safety and discrimination)^(30)^. The original sources and test–retest reliability of C-EAT survey measures are documented in Table 1 when available in the published literature or assessed as part of a related EAT study; test–retest reliability was previously found to be adequate for all measures (test–retest agreement range: 70–90 %). Parental socio-economic status (SES, test–retest *r* = 0·90) and ethnicity/race (test–retest agreement range: 98–100 %) were assessed on the original school-based survey in 2009–2010^(28,31,32)^.

**Table 1:** Description of survey measures

Measure	Survey items or description	Response categories
Food insecurity experiences		
Past year food insecurity	Short form of the US Household Food Security Survey Module^(29)^	Yes/No. A score of 2+ affirmative responses was used to define food insecurity (test–retest agreement = 90 %).
Past month food insufficiency	Two items modified from the short form of the US Household Food Security Survey Module^(29)^: *In the past month, did you ever eat less than you felt you should because there wasn’t enough money for food?* *In the past month, were you ever hungry but didn’t eat because there was not enough money for food?*	If participants responded ‘yes’ to both questions, they were categorised as food insufficient.
Food shopping behaviours		
Shopping online	*In the past month, when shopping for groceries, how often did your household do each of the following activities? Buy groceries online and have them delivered or pick them up outside the store.*	Four Likert-type response options were given for each activity and dichotomised for analysis to distinguish *never/rarely* or *sometimes* from *often* or *always*.
Planning before shopping	Participants were asked how often their household did three activities in the past month: *plan meals before shopping, decide on a set amount of money to spend on groceries,* and *purchase nonperishable food or drinks for a time when you would not be leaving your home.*	Four Likert-type response options were given for each activity and dichotomised for analysis to distinguish *never/rarely* or *sometimes* from *often* or *always* (test–retest agreement for plan meals = 79 %, for decide on set amount of money = 76 %).
Home food availability		
Home food availability	Participants were asked how often five statements were true for the place where they lived (e.g. *apartment, house, dorm room, or other space)* for the majority of the time: *(1) Fruits and vegetables are available (2) Vegetables are part of the dinner meal (3) There is fresh fruit on the counter, table, or somewhere else where I can easily get it (4) There are ready-to-eat vegetables in the fridge and (5) Whole wheat bread is available*	Four response options were given for each statement and dichotomised for analysis to distinguish *never* or *sometimes* available from *usually* or *always* available (test–retest agreement range: 70–80 %)^(8)^.
Eating behaviours		
Frequency of family meals	*During the past seven days, how many times did all, or most, of the people living in your household eat a meal together?*	Six response categories ranging from *never* to *more than seven times* were provided and dichotomised for analysis to distinguish having family meals on 5 + d/week (test–retest agreement = 80 %).
Frequency of eating food from fast-food restaurants	*In the past month, how often did you eat something from the following types of restaurants (include take-out and delivery)?* Participants were asked separately about three categories of fast-food restaurants (i.e. burger-and-fries, pizza, other).	Six response options ranging from *never/rarely* to *one or more times per day* were provided and for analysis were dichotomised to identify frequent consumption on 3 + occasions per week.
Neighbourhood safety and discrimination		
Neighbourhood safety	Participants were asked to respond to two statements regarding whether they felt safe walking in their neighbourhood *during the day* and *at night* ^(41,42)^.	Yes/No.
Discrimination and harassment	Survey items were adapted from established measures^(40,43)^. Participants were asked how often things had happened to them in the past month as part of three statements: *(1) You are treated with less respect or courtesy than other people (2) You are threatened or harassed (3) People have said mean or rude things about you because of your race or ethnic group.*	Five response options were given for each statement and gave the opportunity to indicate a frequency of up to *one or more times a day*. Responses were dichotomised for analysis to distinguish *never* from at least *1 time*.
Personal characteristics of emerging adults		
Demographic characteristics	Self-report of sex, ethnicity/race, employment status, receipt of public assistance, access to personal transportation and living situation. Ethnicity/race (test–retest agreement range: 98–100 %) and parent socio-economic status (SES, test–retest *r* = 0·90) were based on self-report on the school-based survey. The prime determinant of SES was the higher educational level of either parent, with adjustments made for student eligibility for free/reduced price school meals, family public assistance receipt and parent employment status.	Response options for employment included *working full-time, working part-time, temporarily laid off due to the COVID-19 situation (and have no other current work), stay at home caregiver, currently unemployed but actively looking for work, not working for pay (for example, unable to work, student, seasonal worker),* and *other.* Living situations were reported by selecting all that apply from the options *I live alone, my parents, my roommates/friends, my husband/wife, my domestic partner, my child(ren) including any step-children or adopted children, my brothers/sisters,* and *other.*
Influence of COVID-19 on eating and resources needed for supporting health		
Changes in food shopping and eating behaviour	*Please comment on how events related to COVID-19 have influenced your eating behaviors, including how you purchase or prepare food and beverages. What event(s) related to COVID-19 have been the most important to your eating?*	Write-in responses
Providing healthy food for children	When a participant reported having one or more children they were asked: *What has been most helpful to you in providing enough healthy food for your children when schools and many businesses were closed during the COVID-19 infectious disease outbreak? Please tell us about any problems that you had in feeding your children during this time.*	Write-in responses
Resources needed for healthy food	*What resource(s) would most help you to get enough healthy food for yourself and your family during emergencies like the COVID-19 outbreak?*	Write-in responses
Resources needed to support overall health	*Please let us know what other resource(s) would be most helpful to you and your family to support your overall health during emergencies like the COVID-19 outbreak?*	Write-in responses

### Data analysis

#### Quantitative

Frequencies, percentages and *χ* ^2^ tests were initially examined to assess the prevalence of food insecurity in the past year and food insufficiency in the past month across sociodemographic characteristics of emerging adults in the sample. The first study aim was accomplished by focusing on the more common experience of being food insecure at any point over the past year; regression models were used to produce least square mean estimates for measures of food shopping behaviours, home food availability, eating behaviours and neighbourhood safety and discrimination experiences among food-secure and food-insecure emerging adults. Generalised binomial models were used to examine statistical associations of each measure with food security status (*P* values associated with maximum likelihood parameter estimates), and the inverse linked scale option was used to estimate the adjusted prevalence for the measures of interest. Regression models were examined without adjustment and also adjusted for emerging adult sex, ethnicity/race, employment status, household receipt of public assistance and living situation given these variables were identified as correlates of food security in the present analysis or prior research^(8,33,34)^. A third set of models were additionally adjusted for parental SES to build understanding of how the experience of food insecurity may be distinct from the experience of having limited financial resources. As both sets of adjusted models produced similar results and the former is most relevant to intervention design, the model adjusting for SES is not described in detail below. A 95 % confidence level was used to interpret the statistical significance of all probability tests. Analyses were conducted using the Statistical Analysis System (version 9.4, 2015; SAS Institute Inc.).

#### Qualitative

Open-ended survey responses from sixty-two respondents who were food insecure in the past year or food insufficient in the past month (*n* 1 participant was food insufficient in the past month but not food insecure in the past year) were qualitatively coded in order to address the second and third aims of the study regarding how events related to COVID-19 had influenced eating behaviours and what resources were needed. A content analysis was conducted in three stages. The initial stage of coding involved review of all responses by the first author (N.L.) and the generation of several topic areas by which the diverse comments could be categorised. Next, the third author (T.A.) reviewed all comments using the schema developed in the initial coding and assigned each comment to relevant topic categories. Third, there were five coding discrepancies (<1 % of coding decisions) that were identified between the second coder and the initial coder; each of these discrepancies was discussed to resolution. All names in quotes were either removed or changed to protect participant confidentiality.

## Results

### Quantitative findings

#### Associations of past year food insecurity with past month food insufficiency and participant characteristics

Experiencing food insecurity at one or more times in the past year was reported by 28·4 % (*n* 61) of emerging adults and, among this group of food-insecure participants, 41·0 % (*n* 25) reported food insufficiency in the past month (Table 2). Emerging adults who were living with children of their own had elevated prevalence of food insecurity in the past year and past month food insufficiency compared with emerging adults without children. Food insecurity in the past year was also related to parental SES, access to a car or other personal vehicle and receipt of food assistance.

**Table 2:** Prevalence of food insecurity in the past year and food insufficiency in the past month by sociodemographic characteristics of emerging adult respondents to the C-EAT (COVID-19 Eating and Activity over Time) survey in spring 2020*

Characteristics	Sample, *n*	Past year food insecurity† (%)	*P*-value	Past month food insufficiency‡ (%)	*P*-value
Overall	218	28·4		12·0	
Sex			0·34		0·21
Female	153	30·7		14·5	
Male	62	24·2		6·4	
Different identity	3	0		0	
Ethnicity/race§			0·60		0·73
White	79	22·8		11·7	
Hispanic or Latino	42	17·1		7·3	
Asian American	42	18·8		11·9	
Black or African American	31	32·3		16·9	
Mixed or other	23	39·1		18·2	
Parent socio-economic status			< 0·001		0·006
Low	66	34·8		15·4	
Low-middle to middle	75	41·3		18·9	
Upper-middle to high	77	10·4		2·7	
Employment status			0·04		0·21
Working full time	97	19·6		8·3	
Working part time	30	33·3		13·8	
Temporarily laid off or unemployed	59	40·7		19·3	
At-home caregiver/not working for pay	26	26·9		7·7	
Public assistance			< 0·001		0·11
No	169	21·9		10·2	
Yes	49	51·0		18·7	
Living with a child(ren) of your own			0·005		0·02
No	177	24·3		9·8	
Yes	41	46·3		22·5	
Access to a car or other personal vehicle			0·02		
No	36	44·4		20·6	0·11
Yes	182	25·3		10·6	

* C-EAT study participants were originally recruited in Minneapolis–St. Paul, Minnesota public schools in 2009–2010 and 87 % of the sample were living in Minnesota at the time of the spring 2020 survey.

† Food insecurity status was based on responses to the short form of the US Household Food Security Survey Module. Scores of 2+ affirmative responses determined experiencing food insecurity at one or more times in the past year.

‡ Participants were asked ‘In the past month, did you ever eat less than you felt you should because there wasn’t enough money for food?’ and ‘In the past month, were you ever hungry but didn’t eat because there was not enough money for food?’. Food insufficiency in the past month was determined by reporting yes to both questions.

§ Structurally racialised categories labelled by ethnicity/race.

#### Food shopping and eating behaviours by past year food insecurity

Most household food shopping behaviours during COVID-19 were performed by similar percentages of emerging adults who were food-secure throughout the past year and emerging adults who were food insecure in the past year (Table 3). Planning meals and making out a list before food shopping were exceptions with fewer food-insecure emerging adult households engaging in these practices as compared with food-secure households. Food insecurity in the past year was also related to lower prevalence of usually or always having fruits and vegetables available, fresh fruit accessible and whole wheat bread available at home. Food-insecure emerging adults were less likely to report having regular family meals (5+ times/week) and more likely to report eating food purchased from fast-food restaurants on three or more occasions per week. Regardless of food security status in the past year, only a small percentage of emerging adults reported their household bought groceries online and other potentially protective shopping strategies (e.g. purchasing extra nonperishable food or drinks, food budgeting) were engaged in by less than half of food-insecure or food-secure emerging adult households.

**Table 3:** Food shopping behaviours, home food availability and eating behaviours during COVID-19 stay-at-home orders (April–May 2020) by past year food insecurity among emerging adults*

	Unadjusted model 1			Adjusted model 2†		
	Food secure	Food insecure	*P*-value	Food secure	Food insecure	*P*-value‡
Food shopping behaviours (% often/always)						
Plan meals before doing my shopping	50·7	34·4	0·03	50·5	32·3	0·03
Make out a list before doing the shopping	70·6	46·8	0·001	75·8	48·5	< 0·001
Decide on a set amount of money to spend on groceries	21·8	35·5	0·04	17·5	24·1	0·30
Purchase nonperishable food or drinks for a time when you would not be leaving your home	47·2	41·9	0·48	46·9	39·6	0·38
Buy groceries online and have them delivered or pick them up outside the store	10·4	11·3	0·85	6·0	6·9	0·78
Home food availability (% usually/always)						
Fruits and vegetables are available	83·7	67·7	0·01	90·5	71·0	< 0·001
Vegetables are part of dinner	72·4	61·3	0·11	74·6	60·0	0·06
Fresh fruit is accessible	75·8	56·4	0·006	83·3	61·2	0·001
Ready-to-eat vegetables are accessible	67·1	50·0	0·02	68·1	51·3	0·04
Whole wheat bread is available	58·2	45·2	0·08	61·2	40·3	0·02
Eating behaviours						
Family meals (% 5+ times/week)	52·5	39·0	0·08	54·3	33·2	0·02
Frequent fast food intake (% 3+ times/week)	15·7	33·9	0·004	10·8	30·8	< 0·001

* Food insecurity status was based on responses to the short form of the US Household Food Security Survey Module. Scores of 2+ affirmative responses determined experiencing food insecurity at one or more times in the past year.

† Model 2 includes sex, ethnicity/race, employment status, household receipt of public assistance (Supplemental Nutrition Assistance Program and/or Special Supplemental Nutrition Program for Women, Infants, and Children), and living situation (reside with own child *v*. other). Generalised linear models were used to examine statistical associations of each food/eating variable with food security status and the inverse linked scale option was used to estimate adjusted prevalence.

‡ *P* values associated with maximum likelihood parameter estimates for the main effect in adjusted model.

#### Experiences of neighbourhood safety and discrimination by past year food insecurity

Multiple markers of experiencing unsafe neighbourhoods were found to be more prevalent among emerging adults who were food insecure in the past year when differences were examined in unadjusted and adjusted models (Table 4). Being treated with less respect or courtesy than other people, being threatened or harassed and experiencing interpersonal racism in the past month were more often reported by emerging adults who were food insecure compared with those who were food secure throughout the past year. The experience of feeling unsafe when walking in one’s neighbourhood during the day was also more common among those who were food insecure.

**Table 4:** Experiences of neighbourhood safety and discrimination during COVID-19 stay-at-home orders (April–May 2020) by food insecurity among emerging adults*

	Unadjusted model 1			Adjusted model 2†		
	Food secure	Food insecure	*P*-value‡	Food secure	Food insecure	*P*-value‡
Feel unsafe walking in neighbourhood during the day§	6·4	24·2	< 0·001	4·8	17·9	0·002
Feel unsafe walking in neighbourhood at night	41·0	40·3	0·93	33·6	29·7	0·61
You are treated with less respect or courtesy than other people	37·5	61·3	0·001	38·4	61·0	0·01
You are threatened or harassed	9·8	22·6	0·02	6·5	16·4	0·02
People have said mean or rude things about you because of your race or ethnic group	10·0	38·7	< 0·001	6·0	23·4	< 0·001

* Food insecurity status was based on responses to the short form of the US Household Food Security Survey Module. Scores of 2+ affirmative responses determined experiencing food insecurity at one or more times in the past year.

† Model 2 includes sex, ethnicity/race, employment status, household receipt of public assistance (Supplemental Nutrition Assistance Program and/or Special Supplemental Nutrition Program for Women, Infants, and Children) and living situation (reside with own child *v*. other). Generalised linear models were used to examine statistical associations of each safety/discrimination variable with food security status and the inverse linked scale option was used to estimate adjusted prevalence.

‡ *P* values associated with maximum likelihood parameter estimates for the main effect in adjusted model.

§ Race variable was removed from the adjusted models in order to obtain reliable estimates.

### Qualitative findings

The qualitative results of this study focus on the experiences of emerging adults who were food insecure at any point in the past year or food insufficient in the past month prior to participation.

#### Changes in eating behaviour, food preparation and food purchasing among food-insecure emerging adults

Among the comments regarding the influence of COVID-19 on eating behaviours (total *n* 55, Table 5), a common theme was a change in the *amount of food consumed* (*n* 18). Some food-insecure emerging adults reported eating less, whereas others reported eating more than usual due to anxiety, other emotions or boredom. It was also common for food-insecure emerging adults to report *eating fewer meals and/or more snacks* (*n* 14).

**Table 5:** Qualitative themes regarding how events related to COVID-19 influenced the eating and food shopping behaviours of emerging adults who were food insecure in the past year or food insufficient in the past month

Overarching themes of change (number of participants endorsing)	Sub-themes (number of participants endorsing)	*Example quotes*
Changing amount of food consumed (*n* 18)	Eating less food than normal^(8)^	*I don’t eat as much. I work from home now and I make sure the food we have lasts for my kids.*
	Eating more food than normal^(10)^	*I have been eating much more than normal to help cope with the anxiety.*
Eating fewer meals and/or snacking more often^(14)^		*I for sure eat all throughout the day. I honestly have been eating my feelings. When I get irritated, mad, sad, stressed out.*
Changing types of food consumed^(15)^	Eating less fresh food like fruit and vegetables^(5)^	*Since COVID-19, my parents have become unemployed so I spend most of my meal prep money now buying food for the whole family so I haven’t meal prepped for a while and I have just been eating leftover from dinner which tends to be meat and carb heavy with no vegetables since my family doesn’t really eat vegetables.*
	Eating more frozen and non-perishable foods^(5)^	*Eating frozen food which I’ve never done before. Much less vegetables and fruits.*
	Eating more sugary snacks and junk food^(6)^	*I find myself eating more than the normal 3 times a day. I always want unhealthy snacks. I have been purchasing junk food.*
Changing frequency of eating food from restaurants^(16)^	Eating more food from restaurants^(9)^	*They have closed the YWCA on Lake Street that I normally go to everyday to exercise and run. I also like going to Chipotle a lot, but with social distancing and wait times I feel like going to places like McDonald’s or like Arby’s due to impatience and fear of getting infected around others. McDonald’s is the very less healthy substitute for what’s happening.*
	Avoiding food from restaurants^(5)^	*I can’t go to a restaurant at all which I like to do twice a month.*
Preparing more food at home^(11)^		*I work in a healthcare setting and COVID-19 has caused me to be more mindful when purchasing and touching foods on shopping trips, etc. I am now doing most of my cooking at home and preparing meals for myself compared to my pre COVID-19 buying mostly take out eating habits.*
Changing patterns of food shopping^(18)^	Frequency of shopping^(5)^	*It has definitely made me and my family eat more careful and how we spend our money, to having to make sure everything last the family so we don’t have to run out to the grocery store so much.*
	Quantities of food purchased and shortages^(9)^	*I have been purchasing more food each time I go to the grocery store including many more non-perishables than usual.*

Changes in the *types of food consumed* was another commonly reported theme (*n* 15). Some food-insecure emerging adults reported eating more healthy foods for reasons such as boosting their immunity; however, most who mentioned this topic reported shifts towards eating less fresh food, more frozen and non-perishable foods, and more sugary snacks and junk food.

Events related to COVID-19 led a number of food-insecure emerging adults to *eat more food prepared at home* (*n* 11) and for some to also have more meals that were prepared for sharing as a household. In correspondence with eating more food prepared at home, a number of emerging adults reported *changing patterns of food shopping* (*n* 18). Food shopping frequency was reported by some emerging adults to increase; however, there was much variability with others reporting they changed how they shop to reduce their number of trips to the store. While shortages at grocery stores made it more difficult to purchase desired quantities of food, a few emerging adults reported purchasing more non-perishables when possible.

There was variability in the comments of emerging adults regarding *changing the frequency of eating food from restaurants* (*n* 16). Fears about getting sick at the grocery store led some food-insecure emerging adults to eat more take-out food from restaurants, whereas others had concerns about the safety of restaurant food. Among emerging adults who reported eating out, traditional fast-food restaurants were perceived by some to be the safest option.

#### Resources needed by emerging adults to get enough healthy food

When asked about resources needed to help in getting enough healthy food for yourself and your family (Table 6), most of the comments made by emerging adults who were parents (total *n* 18) and the overall sample of food-insecure young people (total *n* 48) focused on a desire to receive *food assistance benefits,* including needs for a larger amount of benefits, having food delivery or pick-up options, and access to food at food pantries (parent *n* 10, overall *n* 19). Emerging adults experienced challenges with these resources due to limited eligibility for benefits as well as troubles with accessing food (e.g. for their family at food pantries, for their children at schools) that would serve the needs of their culturally diverse families.

**Table 6:** Resources most helpful or needed to get enough healthy food for feeding children (named by parents) and nourishing yourself and household (named by overall sample): reported by emerging adults who were food insecure in the past year or food insufficient in the past month

School food programmes and food shelves with options for culturally diverse households Larger amounts of benefits provided by Supplemental Nutrition Assistance Program and the Special Supplemental Nutrition Program for Women, Infants, and Children Vouchers and reduced prices for healthy foods like fruits and vegetables Having options for purchasing online with food assistance benefits and having food delivery or pick-up services Store policies with adaptations for single parents Frequent restocking of food items in stores Planning of meals and snacks Eligibility for stimulus and relief funds for persons with diverse living situations (e.g. households with no children)

*Relief funds and money* (overall *n* 15) were also among the most common responses from food-insecure emerging adults with regard to needed resources. Emerging adults mentioned stimulus checks, relief funds, unemployment benefits, inadequate household income and the cost associated with healthy food. Eligibility for financial resources was particularly challenging for emerging adults who were single and did not have children of their own.

Resources were needed by emerging adults to address challenges with safely *accessing food at retail stores* (parent *n* 2, overall *n* 9). Food-insecure emerging adults raised concerns relating to store policies that did not allow children to enter, out-of-stock items, inadequate cleaning procedures and lack of opportunities for ordering food online. In alignment with the quantitative findings, one participant specifically mentioned discrimination (i.e. fearing to go out because of their race) and another described worries about having to go to the store to purchase food with food assistance benefits.

#### Resources needed to support overall health among food-insecure emerging adults

When asked more generally about resources needed to support their overall health and the health of their family (Table 7), most of the comments (total *n* 31) made by food-insecure emerging adults again focused on needs for *money* (*n* 11), *food assistance* (*n* 8) and *challenges with food access* (*n* 6). In particular, several participants mentioned the need for financial assistance to pay for rent. *Health care* was also a challenge for food-insecure emerging adults with comments (*n* 6) made regarding the cost, need for more mental health resources and the desire to have universal care and virtual care (e.g. telehealth) options.

**Table 7:** Resources needed to support the overall health of emerging adults and their families who were food insecure in the past year or food insufficient in the past month

Food assistance in the form of food boxes that include resources based on the number of residents Community-based food shelves and pantries Retail store policies that limit food hoarding Access to options for purchasing food from an online grocery store More relief funds for single adults with no children Rent assistance and unemployment benefits Mental health resources Access to virtual medicine (e.g. video-based clinic appointments) Universal health care

## Discussion

This study is one of the first to our knowledge to examine food insecurity and intersecting barriers to health during the US outbreak of COVID-19 and was specifically designed to help inform the development of policies and services for emerging adults. The existing literature on food insecurity during the outbreak has focused on the high prevalence and disparities among population-based samples of adults, households with children and university students^(17,23–25)^. Results of the present study align with and extend prior research in finding that food insecurity is a prevalent problem among diverse emerging adults and common needs during the outbreak for resources such as support for food delivery costs, improved stocking of food in retail stores and increased food assistance benefits^(17,23,25)^. As structural racism has resulted in a disproportionate burden of COVID-19 for Black, Indigenous and people of colour^(35–37)^, the present study also included a novel focus on the co-occurrence of food insecurity with neighbourhood safety concerns and exposure to interpersonal forms of discrimination that may further limit access to healthy food. The findings indicate that emerging adults who were food insecure in the past year had poorer access to healthy foods at home, more frequent fast-food restaurant meals and more experiences of discrimination and feeling unsafe during the initial months of the COVID-19 outbreak when compared with emerging adults who were food secure. While the influence of COVID-19 on eating behaviour resulted in some health promoting shifts (e.g. preparing more food at home), most of the changes described by food-insecure emerging adults are likely to have negative health consequences. The results regarding unhealthy changes in food purchasing and eating are in line with other recent studies and emphasise the unique challenges faced by emerging adults during a public health emergency^(24)^.

Results of the present study indicate that a majority of emerging adults have not recently made use of food shopping practices that could help them to more easily manage their resources for food and safety when shopping. Although the use of most food shopping practices was unrelated to food security status, practices such as planning meals before shopping and deciding on a set amount of money to spend could be particularly helpful for young people to manage their food needs and limit the time they need to spend in retail food stores. The practice of buying groceries online could also benefit emerging adults when store policies do not allow them to enter with children or they have a concern about potentially being exposed to COVID-19 while shopping. At the time of the C-EAT survey, only a small percentage of emerging adults reported buying groceries online; however, most Minnesota retailers did not yet have available the option for food assistance benefits to be used for purchasing groceries online. The recent expansion of the Supplemental Nutrition Assistance Program Online Purchasing Pilot in Minnesota and other states may lead to changes in the prevalence of this practice among food-insecure emerging adults if they are made aware of the new option and strategies for planning needed purchases around available time windows for pick-up and delivery^(22)^. Prior research has demonstrated there may be discomfort around making online purchases of nutrient-dense food items that are perishable (e.g. fruits and vegetables, meat), and thus strategies may also be needed for optimally using online options to purchase these foods^(38)^.

Another unique contribution of the present study relates to building understanding of how food shopping, food preparation and eating behaviours of food-insecure emerging adults have been influenced by the COVID-19 outbreak. The findings may provide valuable guidance to organisations working to provide food assistance and health education during the current outbreak and future public health emergencies. Many emerging adults reported worries about becoming infected with COVID-19, but, in alignment with the state of confusion about health recommendations at the start of the outbreak, their worries led to different types of shifts in eating behaviour. Preparing more food at home and purchasing more non-perishable foods were common responses to worries about COVID-19. It was also common for emerging adults to report eating take-out food from restaurants and a shift to purchasing more often from traditional fast-food restaurants that provide drive-thru services. While some emerging adult respondents had fears about getting infected with COVID-19 as a result of poor food safety practices in fast-food restaurants, others were more concerned with getting infected as a result of being around other people in a crowded restaurant or grocery store. Findings from the present study suggest there is a need for clear and consistent messaging with a broad reach to inform young people about how to safely manage the purchasing of healthy food and meal planning during an infectious disease pandemic. For example, it may be helpful for agencies involved in providing food assistance to also have a role in educating young people on safety practices such as wearing a mask inside stores and restaurants, washing fruits and vegetables and thorough hand washing. Government programmes that focus on providing nutrition education to low-income populations (e.g. Supplemental Nutrition Assistance Program Education, Expanded Food and Nutrition Education Program) may be particularly well positioned to provide this vital information.

The findings of the present study build on what is known about linkages between experiences of interpersonal racism and food insecurity among ethnically/racially diverse emerging adults. The small number of prior investigations regarding this relationship have focused on households with children^(13,14)^. For example, research involving Latinx and Black/African American mothers of young children in Philadelphia found that experiences of discrimination from police, courts and in workplaces were associated with a greater likelihood of being food insecure^(13)^. Discrimination in workplaces, schools and courts as examined by existing studies might plausibly be linked to greater food insecurity by pathways involving higher rates of incarceration, lower wages, lower rates of promotion and poorer job security. The emerging adult participants who reported experiencing racism in the month prior to the C-EAT survey were not asked about the specific settings in which people said mean or rude things to them because of their race or ethnic group, but many of the same pathways are likely to hamper access to healthy food for these young people and members of their household. Although most participants in the present study were not financially responsible for children of their own and 40 % were not independently responsible for housing expenses, it is likely that experiences of racism had a significant impact on the wages and job security of emerging adult participants and thus the adequacy of their household income. Additional research is needed to explore whether experiences of interpersonal racism in grocery stores and restaurants may play a role in food access and possibly compound the consequences of structural racism on access to large grocery stores and supermarkets^(39)^.

Study weaknesses to be taken into account in interpreting the findings include the limited generalisability of responses primarily coming from a convenience sample of emerging adults in one Midwestern state and the brevity of survey measures. It is a limitation that participants completed only the short form of the US Household Food Security Survey Module; although this tool is validated and ideal for limiting participant burden during a stressful time, it does not allow for assessing the most severe levels of food insecurity or conditions of children in the household. Participants completed measures of unsafe neighbourhood conditions and exposure to discrimination that were adapted from established tools, but the measures were very brief (e.g. captured only discrimination that involved having someone say mean or rude things about you). The C-EAT survey did not address all possible forms of discrimination and safety concerns and did not specifically assess the ways that these problems might influence one’s ability to access healthy food^(40–43)^. Also, because the measures of food shopping behaviours and experiences of neighbourhood safety and discrimination were not included in the 2018 survey and the 2018 survey included only a two-item measure of experiencing food insufficiency in the past year, the study design was not ideal for examining changes from before to after the US outbreak of COVID-19. The measure of SES was validated by parental report at baseline of the study and incorporated information on parental education, parental employment and receipt of public assistance in order to help prevent misclassification of resources^(44)^. Despite the established nature of the measure, it is important to acknowledge that SES may improve or decline as young people transition from adolescence to adulthood. The small size of the sample and variability of individual experiences also led to low numbers for some of the qualitative themes identified by the analysis and thus make the results more challenging to interpret.

Strengths of the present study include the combined use of quantitative and qualitative data, breadth of food behaviours examined and the sociodemographic diversity of emerging adult participants. The timely collection of surveys from young people impacted by emergency stay-at-home orders will help to fill an urgent need for data to inform health protection policies and services. In particular, the analysis of open-ended comments provides important context for understanding the challenges faced by emerging adults who responded to survey measures in a manner indicative of experiencing food insecurity. It will be important for additional studies to examine the impact of stay-at-home orders on food insecurity in other population-based samples of emerging adults; however, the present study provided valuable insights into the experiences of ethnically/racially diverse young people and was inclusive of persons living alone, persons living with their parents and others who were living with families of their own.

In conclusion, the results described here demonstrate that emerging adults are experiencing many barriers to accessing food and maintaining healthful eating patterns during the US outbreak of COVID-19. The national prevalence of food insecurity in the past 30 d was 5·5 %, and 2·3 % of US households experienced very low food security in December 2019 prior to the outbreak of COVID-19^(10)^. The much higher prevalence of 12 % of emerging adults reporting food insufficiency in the past 30 d during the COVID-19 outbreak is of great concern. Research building on the present study is needed to guide the ongoing response to food insecurity in areas impacted by COVID-19 outbreaks and as part of future public health emergencies^(7)^. It will be important for follow-up studies to evaluate how the needs of emerging adults may change as communities continue to cope with COVID-19 and what challenges are encountered after stay-at-home orders are lifted. The role of racism in food insecurity is complex. The results reported here suggest it would be worthwhile to further use more in-depth measures to assess exposure to racism and explore the perceptions of emerging adults regarding how various forms of racism influence their shopping habits and ability to obtain healthy food. It could likewise be informative to comprehensively investigate experiences of racism within settings such as schools, food pantries and sites that administer nutrition assistance programmes and how they might influence food access for emerging adults. Efforts to simplify the process of accessing food assistance for emerging adult populations should be evaluated along with messages about potentially beneficial food purchasing practices (e.g. planning meals before shopping).

Despite the need for additional research, the results of the present study emphasise the importance and urgency of supporting ongoing efforts to expand food assistance benefits, benefit flexibilities and relief funds in order to provide emerging adults with the tools they need to maintain their health in times of public health emergencies. The response of the US Department of Agriculture Food and Nutrition Service to COVID-19 has begun to address many of the challenges that were raised by emerging adults as part of this study, and nutrition professionals will have an important role in ensuring that programme changes are communicated broadly and evaluated^(22)^. Access to adequate benefits through federal programmes has been a particular challenge that needs to be monitored for emerging adults who do not have a spouse or children and may be living alone or with other adults who are employed. Additionally, the results strongly suggest the need for public health efforts to reduce barriers to the safe purchasing of healthy foods and ensure local nutrition programmes provide culturally acceptable food options for all community members in need. The option to use food assistance benefits to purchase food online and have the food delivered or available for pick-up outside the store was identified as very desirable, and thus it will be important for nutrition professionals to support, help to evaluate and help to educate emerging adults about the Supplemental Nutrition Assistance Program Online Purchasing Pilot^(22)^. Findings of the present study further emphasised the need for nutrition professionals to take an active role in addressing racism and neighbourhood safety given the strong relationships between these public health problems and food insecurity. Safety with regard to avoiding exposure to COVID-19 when purchasing food and meal planning are yet more challenges that can be addressed by the work of nutrition professionals in providing guidance on how to safely manage the purchasing and preparation of healthy food. The diversity of experiences reported by young people as part of this study further suggest that nutrition professionals will need to be responsive to unique challenges and should take time to ask about and listen to how their clients are coping with the health risks and food access barriers posed by COVID-19.
